# The Impact of Politicization on Adolescent Medicine Care: A Rapid Review

**DOI:** 10.1007/s40124-026-00379-9

**Published:** 2026-07-08

**Authors:** Laura Stamm, Breanna De Leon, Da’Jon M. Stoudemire, Milena E. Insalaco, Ahona Shirin, Madalayne Martin-Olenski, Carley Daly, Kristefer Stojanovski

**Affiliations:** 1 Department of Medicine, Health Humanities and Bioethics, University of Rochester, Rochester, NY USA; 2 Department of Behavioral and Community Health Sciences, School of Public Health, University of Pittsburgh, Pittsburgh, PA USA; 3 Celia Scott Weatherhead School of Public Health and Tropical Medicine, Partners for Advancing Health Equity, Tulane University, New Orleans, LA USA; 4 Department of Surgery, University of Rochester, Rochester, NY USA; 5 Department of Medicine, University of Rochester, Rochester, NY USA; 6 School of Medicine and Dentistry, University of Rochester, Rochester, NY USA; 7 Department of Social, Behavioral and Population Sciences, Celia Scott Weatherhead School of Public Health and Tropical Medicine, Tulane University, New Orleans, LA USA

**Keywords:** Adolescent medicine, Health policy, Politicization, Right to health, Reproductive care, Gender-affirming care

## Abstract

**Purpose of Review:**

Increased politicization of health care, exemplified by bans on gender-affirming and reproductive care, has significantly impacted adolescents’ ability to receive safe care. We conducted a rapid review of evidence examining politicized health legislation’s impact on adolescents’ right to health, focusing on reproductive, gender-affirming, and eating disorder care.

**Recent Findings:**

Politicization of care has impacted adolescents’ right to health by creating financial barriers and changes in geographic access. For example, state legislative ban on abortion and gender-affirming care have created care deserts in which adolescents are forced to travel to receive care. These changes have resulted in delays in care, loss of bodily autonomy, and negative impacts on physical and mental health.

**Summary:**

The findings of this rapid review have important implications for adolescent medicine clinical practices. Providers caring for adolescents require institutional support and resources to address increased patient psychosocial concerns and potentially higher patient volume as a result of restrictive legislation. Ultimately, policy advocacy is necessary to change legislation disrupting adolescents’ right to health.

**Supplementary Information:**

The online version contains supplementary material available at https://doi.org/10.1007/s40124-026-00379-9.

## Introduction

The increased politicization of medical care in the United States has disproportionately impacted adolescent medicine [1, 2]. Bans on medical care, disinformation, and a challenging sociopolitical environment all affect the supposed private relationship between patient and provider [3, 4]. Alongside state legislation, shifting institutional policies often make it difficult for providers to deliver evidence-based care that aligns with their patients' needs. The inability to access desired care impacts adolescents’ mental and physical health [5–7]. While many commentaries have discussed the impact of politicization on adolescent medicine, there has been no major review of the empirical evidence [8–10]. Moreover, most studies on the topic of politicization have looked at one aspect of care (e.g., gender-affirming care) instead of addressing the wider spectrum of care provided in adolescent medicine.

The Supreme Court’s 2022 *Dobbs v. Jackson Women’s Health Organization* ruling overturned *Roe v. Wade* (1973) and *Planned Parenthood v. Casey* (1992) and eliminated the constitutional right to privacy that protected a person’s freedom to access abortion care. The ruling returned control to the states, which led to legislation restricting reproductive care, including outright abortion bans, gestational limits, and parental notification laws [2, 11]. This fractured national landscape has disrupted adolescent medicine care by creating concerns about privacy and confidentiality and the need for patients to travel to receive care [12]. Given the increased challenges young adults face to accessing contraception and abortion services, adolescent medicine providers play a key role in addressing adolescent patient needs through prescription and referral [13, 14]. However, many providers are now unable to do so due to lack of proper training, institutional support, and/or legal ability to provide referral [15, 16].

In conjunction with post-*Dobbs* state legislation, conservative states began introducing state legislation banning gender-affirming care for those under 18 in 2021; Arkansas was the first state to do so with the passing of House Bill 1570 [17, 18]. Twenty-seven states now have laws restricting care, while 14 states have passed shield law legislation intended to protect access to care [19]. Restrictive legislation contradicts the guidance of every major medical association and prevents transgender and gender-diverse youth from accessing evidence-based treatments for gender dysphoria [8, 20, 21]. These restrictions have had devastating effects on transgender and gender-diverse youth, as many families have now been forced to travel or relocate entirely to access care [22]. Their access has also been affected by a strained workforce; adolescent medicine trainees have uneven access to gender-affirming care education, many providers have relocated to safer states, and the majority of providers and trainees experience great distress due to political attacks on the care they provide [23–25].

The politicization of adolescent medicine care, exemplified by Post-*Dobbs* abortion care and gender-affirming care legislation, has infringed upon adolescents’ right to health [26]. As first described by the World Health Organization in 1946, the right to health asserts that each person has the right to the highest attainable standard of health [27]. When applied to care delivery, the right to health is measured by care availability, accessibility, acceptability, and quality (3AQ) [28]. In this rapid review, we sought to understand the impact of politicization, defined by care-related policy and legislation, on adolescents’ right to health. We considered how legislation impacted reproductive, gender-affirming, and eating disorder care for adolescents and young adults across the United States.

## Methods

### Search Strategy

We reviewed studies aligned with Preferred Reporting Items for Systematic review and Meta analysis (PRISMA) criteria [29]. Medical Subject Headings (MeSH) terms were deployed across databases in February 2026 with the support of health sciences librarians. MeSH terms for the following search terms were used: legislation, policy, regulation, politic; youth, child, adolescent; gender-affirming care, gender diverse; eating disorders, anorexia, bulimia; and abortion. We also included terms to limit the search to 2006 or later, English only, United States, and empirical studies. The full search is available in Supplementary Table 1. The databases searched included Scopus (*n* = 675), PubMed (*n* = 1,590), Web of Science (*n* = 84), Global Health (*n* = 38), and CINAHL Complete (*n* = 337). These studies (*n* = 2,724) were imported into Covidence, a web-based software that assists with data screening and extraction [30]. Covidence identified and subsequently removed 454 duplicates in the literature search, and 11 duplicates were removed manually, resulting in 2,259 articles for title and abstract screening.

### Inclusion and Exclusion Criteria

Inclusion criteria were as follows: (1) include adolescent, 10–25 years of age, patient/client population; (2) included a policy, regulation, rule that was politicized in nature (3) outcome included the right to health (accessibility, availability, acceptability, and quality of care) (4) examined how the policy/rule/regulation was associated with the right to health outcome; (5) results presented must have included findings for those 10–25 years of age; (6) peer-reviewed empirical articles; (7) written in English; (8) within the last 20 years (2006–2026), and (9) included USA populations. If articles did not align with any one of the above, they were excluded.

### Screening and Data Extraction

**Fig. 1:**
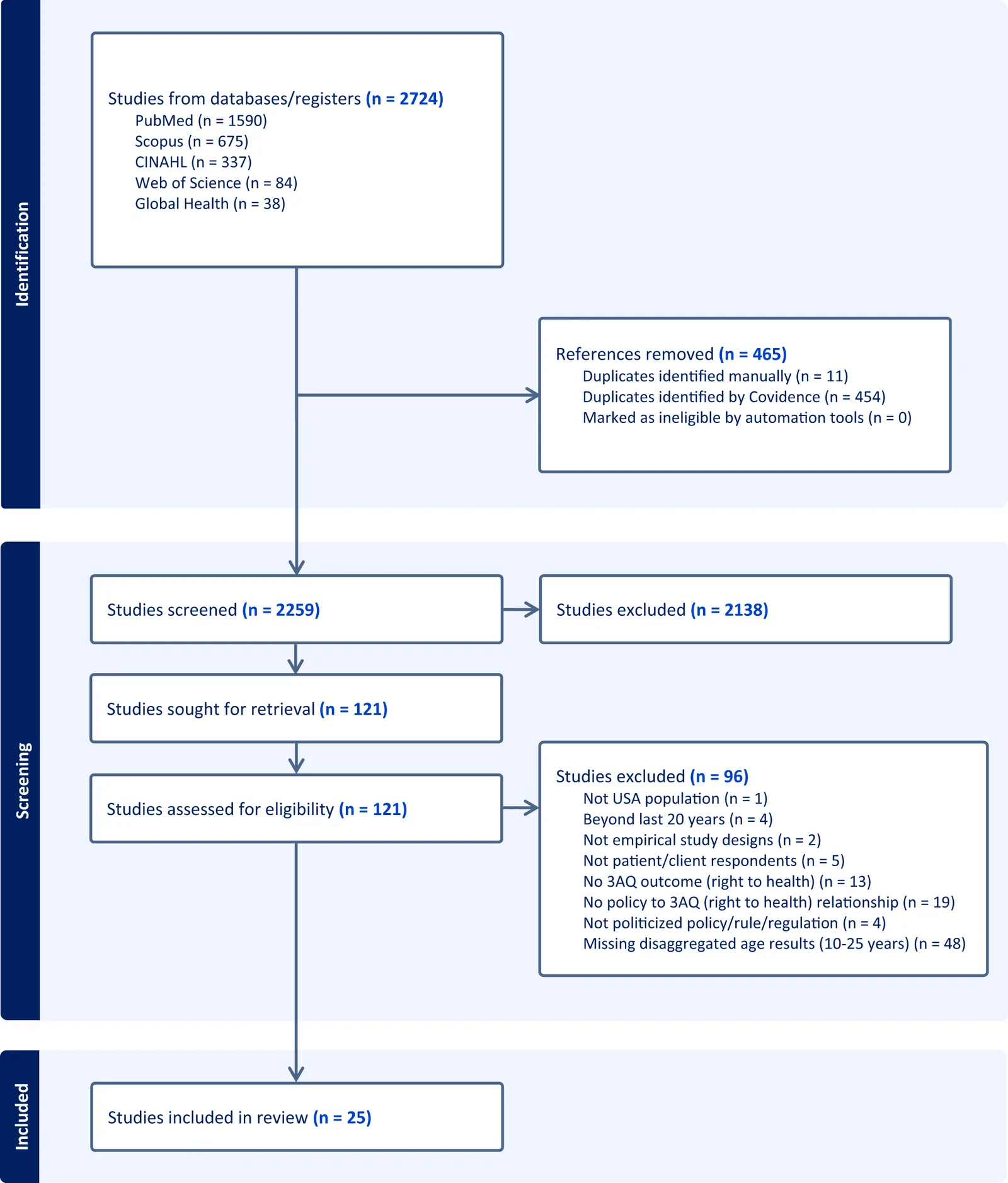
PRISMA flow diagram

Eight reviewers (MEI, MMO, CD, BDL, AS, DMS, LS, KS) conducted title and abstract screening (phase one) and full-text review (phase two) independently and in duplicate. Consensus in both screening phases was achieved through a third reviewer (LS or KS). We first screened five articles, then met to resolve conflicts and ensure alignment across reviewers. After the meeting, we screened another five articles and no additional conflict arose. Of the 2,259 articles screened, 2,138 were deemed ineligible for inclusion in the title and abstract phase. Reasoning for exclusion at this stage is not required under PRISMA guidelines [29]. Of the remaining 121 articles, 96 did not meet the full-text inclusion criteria; 48 were removed due to lack of disaggregated results on our age population, 19 did not examine the relationship between the policy and 3AQ, and 13 were missing our 3AQ outcome. The remaining reasons for exclusion are shown in the PRISMA diagram (Fig. 1). A total of 25 studies were included for data extraction.

With advances in artificial intelligence, particularly natural language processing, we conducted data extraction with the assistance of Elicit [31]. Elicit is an artificial intelligence tool designed to analyze scientific research articles, which has been evaluated to be useful and rigorous with human verification included in the process [32–35]. Each article PDF was uploaded into Elicit for data extraction. Study design, location, sample size, participant demographics, the right to health outcome and its measurement, the policy and its measurement, as well as the results examining the relationship between the policy and right to health were extracted by Elicit through prompts composed by the research team. An example prompt used to extract the results was “Provide the relationship between the exposure [policy] and the outcome, providing statistical findings (for quantitative studies) or themes (for qualitative studies).” The extracted data includes the requested information, indicates the location in the article from which the data is extracted, and provides reasoning for the responses to the queries. The extracted data were downloaded into an Excel file. Reviewers assigned to that paper extracted the information from every study, resolved any conflicts in extraction, and incorporated missing details needed by adding additional information during human verification and review of all the articles. We were particularly focused on results that aligned with the age group as part of our inclusion criteria (10–25 years of age).

### Quality Assessment

Articles in the extraction phase were assessed for quality by two human reviewers. Studies were assessed with their respective checklist. This included the 7-item Joanna Briggs Critical Appraisal Checklist for Analytic Cross Sectional Studies [36], the 11-item Joanna Briggs Critical Appraisal Checklist for Cohort Studies [37], the 9-item Joanna Briggs Critical Appraisal Checklist for Quasi-Experimental Studies [38], the 10-item Critical Appraisal Skills Programme checklist for qualitative studies [39] and the 10-item Mixed Methods Appraisal Tool [40]. Studies were rated “Poor” (< 75%), “Fair” (75–90%), or “Good” (90–100%) based on the percentage of checklist questions answered affirmatively. Out of the 25 articles, 12 were rated as “good” quality, nine as “fair”, and four as “poor.” The extracted data on study characteristics for the 25 articles are found in Table 1, and the extracted results and quality ratings are in Table 2.

**Table 1:** Study characteristics

Study ID	Study design	Geographic location (USA)	Total sample size (*n*)	Adolescent sample size (*n*)	Adolescent age groups (*n*)	Adolescent gender identity (*n*)	Adolescent sexual orientation (*n*)
Baum et al., 2016 [42]	Qualitative	Texas	Total: 20	Adolescent: 12	18–21: 6 22–25: 6	Women: 12	Unspecified
Biggs et al., 2025 [65]	Cross-sectional	National	Before Dobbs 2021–2022 Total: 6,982 After Dobbs 2023 Total: 3,561	Before Dobbs 2021–2022: Adolescent: 1,632 After Dobbs 2023 Adolescent: 886	Before Dobbs 2021–2022 15–17: 175 18–19: 60 20–24: 375 25–29: 1,022 After Dobbs 2023 15–17: 196 18–19: 56 20–24: 207 25–29: 424	Female assigned at birth: 886	Not disaggregated for adolescents
Borah et al., 2023 [51]	Cross-sectional	National	Total: 297,600	Adolescent: 297,600	Range: 13–17	Transgender: 297,600	Unspecified
Brandon-Friedman et al., 2025 [63]	Qualitative	Indiana	Total: 46	Adolescent: 22	Range: 12–18 Mean: 14.8 SD: 1.98	Demiboy: 1 Man/Male: 2 Trans men: 7 Trans women: 4 Identity not listed: 1 No answer: 1	Pansexual: 5 Queer: 3 Identity not listed: 2 I do not know: 3
Carter et al., 2026 [62]	Qualitative	Florida	Total: 105	Adolescent: 105	Range: 18–26	Nonbinary, Agender, Gender Nonconforming, or Genderfluid: 34 Trans man: 25 Transmasc or Nonbinary Transmasc: 13 Trans woman: 27 Transfem or Nonbinary transfem: 6	Bisexual/Pansexual: 50 Queer:24 Gay/Lesbian: 16 Heterosexual/Straight: 7 Asexual: 7 Questioning: 1
Crowe et al., 2025 [54]	Quasi-experimental	Texas	IUD Total: 14,693	Adolescent: 8,379	IUD Mean: 25–29	Women: 8,379	Unspecified
Ehrenreich & Marston, 2019 [57]	Qualitative	Utah	Total: 20	Adolescent: 13	18–19: 2 20–24: 2 25–29: 9	Women: 13	Unspecified
Fuentes et al., 2016 [43]	Qualitative	Texas; New Mexico	Total: 23	Adolescent: 15	18–25: 15	Women: 15	Unspecified
Fuerst et al., 2024 [58]	Cohort	Oregon	Pre-Dobbs Total: 61,832 Post-Dobbs Total: 10,972	Pre-Dobbs Adolescent: 24, 557 Post-Dobbs Adolescent: 24, 557	Pre-Dobbs Less than 18: 3,239 18–25: 21,318 Post-Dobbs Less than 18: 548 18–25: 3,6	Pre-Dobbs Women: 24,557 Post-Dobbs Women: 4,236	Unspecified
Garcia & Crosby, 2020 [49]	Qualitative	Oregon	Total: 25	Adolescent: Unspecified	Mean: 27.6 SD: 6.2	Transgender Women: 25	Not disaggregated for adolescents
Jerman et al., 2017 [46]	Qualitative	Michigan; New Mexico	Total: 29	Adolescent: 21	18–19: 2 20–24: 11 25–29: 8	Women: 21	Unspecified
Johnson et al., 2025 [64]	Qualitative	National	Total: 39	Adolescent: 17	18–19: 1 20–24: 8 25–29: 8	Unspecified by age	Not disaggregated for adolescents
Kimport & Weitz, 2025 [61]	Qualitative	Multi-state	Total: 30	Adolescent: Unspecified	Range: 18–43	Cisgender women: Unspecified	Unspecified
King et al., 2025 [14]	Mixed-methods	National	Summer 2022 Total: 638 Summer 2023 Total: 596	Summer Adolescent: 638 Summer 2023 Adolescent: 596	Summer 2022 Range: 14–24 Mean: 19.9 < 18: 109 ≥ 18: 529 Summer 2023 Range: 14–24 Mean: 19.9 < 18: 122 ≥ 18: 474	Summer 2022 Female: 294 Male: 258 Nonbinary/Order: 86 Summer 2023 Female: 301 Male: 215 Nonbinary/Order: 80	Unspecified
Koenig et al., 2023 [56]	Cross-sectional	Multi-state	Clinical Chart Total: 6,027 Survey Total: 1,600	Clinical Chart Adolescent: 4,576 Survey Adolescent: 1,279	Clinical Chart < 18: 30 18–24: 1,458 25–34: 3,088 Survey < 18: 8 18–24: 448 25–34: 823	Unspecified	Unspecified
Kuroki, 2025 [48]	Cross-sectional	National	Total: Unspecified	Adolescent: Unspecified	18–19: Unspecified 20–24: Unspecified 25–29: Unspecified	Woman: Unspecified	Unspecified
Ralph et al., 2024 [53]	Cross-sectional	National	Dec 2021 – Jan 2022 Total: 7,016 Jun 2023 – Jul 2023 Total: 7,148	Dec 2021 – Jan 2022 Adolescent: 1,639 Jun 2023 – Jul 2023 Adolescent: 1,780	Dec 2021 – Jan 2022 15–17: 175 18–24: 439 25–29: 1,025 Jun 2023 – Jul 2023 15–17: 363 18–24: 536 25–29: 881	Dec 2021 – Jan 2022 Female assigned at birth: 1,639 Jun 2023 – Jul 2023 Female assigned at birth: 1,780	Not disaggregated for adolescents
Ramesh et al., 2016 [59]	Quasi-experimental	Illinois	Before Total: 320 Adolescent: 320 After Total: 311 Adolescent: 311	Before Adolescent: 320 After Adolescent: 311	Before 12–15: 85 16–17: 235 After 12–15: 66 16–17: 245	Before Women: 320 After Women: 311	Unspecified
Roberts et al., 2016 [45]	Cohort	Utah	Total: 500	Adolescent: Unspecified	Mean: 25.6	Woman: Unspecified	Unspecified
Strasser et al., 2025 [55]	Mixed-methods	National	Quantitative Total: Unspecified Qualitative Jul 2022 Total: 638 Qualitative May 2023 Total: 596	Quantitative Adolescent: Unspecified Qualitative Jul 2022 Total: 638 Adolescent: 638 Qualitative May 2023 Adolescent: 596	Quantitative: Unspecified Qualitative Jul 2022 Range: 14–24 < 18: 109 ≥ 18: 529 Qualitative May 2023 Range: 14–24 < 18: 122 ≥ 18: 474	Qualitative Jul 2022 Female: 294 Male: 258 Nonbinary/Other: 86 Qualitative May 2023 Female: 301 Male: 215 Nonbinary/Other: 80	Unspecified
Upadhyay et al., 2014 [52]	Cohort	Multi-state	Total: 956	Adolescent: Unspecified	15–17: 173 18–24: 346 25–34: 365	Woman: 956	Unspecified
Vear et al., 2023 [47]	Cross-sectional	National	Total: 638	Adolescent: 638	Mean: 19.8 Range: 14–24	Female: 294 Male: 258 Nonbinary: 50	Unspecified
Washburn et al., 2024 [50]	Longitudinal	Multi-state	Total: 2,420	Adolescent: 2,420	Range: 14–26	Female assigned at birth: 1,662 Male assigned at birth: 758	LGBTQ+: 868 Not LGBTQ+: 1,552
White et al., 2016 [44]	Qualitative	Alabama	Total: 25	Adolescent: 17	19–24: 11 25–29: 6	Women: 17	Unspecified
White et al., 2026 [60]	Quasi-experimental	Multi-state	Before Total: 44,750 After Total: 36,478	Before Adolescent: 29,998 After Adolescent: 24,38	Before < 18: 1,016 18–24: 16,256 25–29: 12,726 After < 18: 751 18–24: 13,068 25–29: 10,566	Unspecified	Unspecified

Note. Table content reflects the original wording used in each study

**Table 2:** Extraction and quality assessment

Study ID	Politicization measurement	3AQ outcome	Study findings	Result themes*	Quality assessment rating
Baum et al., 2016 [42]	Texas House Bill 2	Accessibility & Availability: Abortion Care	The implementation of restrictive abortion laws in Texas led to significant barriers for women seeking abortion services, including informational, cost, and logistical challenges.	1, 3, 5	Good
Biggs et al., 2025 [65]	Dobbs v Jackson Women’s Health Organization	Accessibility: Abortion Care	The number of over-the-counter access to abortion medication increased following the Dobbs decision (36.0% before to 42.5% after Dobbs; *p* < 0.001).	4	Good
Borah et al., 2023 [51]	State enacted legislation, executive actions, or other policies restricting health care for transgender youths	Accessibility: Gender-Affirming Care	National median drive times to abortion clinics significantly increased after restrictions were implemented, rising from 0.51 to 0.99 h (*p* < 0.001). The largest increases were observed in Florida (+ 8.5 h), Texas (+ 6.7 h), and Utah (+ 5.0 h), while states without restrictions showed little change; in contrast, median drive times in restrictive states increased from 0.8 (0.4–1.5) hours to 5.3 (3.2–7.6) hours.	1, 2, 5	Fair
Brandon-Friedman et al., 2025 [63]	Indiana Senate Enrolled Act 480	Accessibility: Gender-Affirming Care	Laws banning gender-affirming medical interventions for minors significantly impacted gender-diverse youth and their families by limiting access to care.	3, 4, 5	Good
Carter et al., 2026 [62]	Florida Senate Bill 254	Accessibility & Availability: Gender-Affirming Care	Transgender and nonbinary young adults faced significant barriers in accessing gender-affirming medical treatment due to restrictive legislation, leading to gaps in access, and delays in care.	3, 4, 5	Good
Crowe et al., 2025 [54]	Texas House Bill 2	Accessibility & Availability: Abortion Care, Contraception	Restricted abortion access significantly increased demand for IUDs, with no effect on vasectomies.	1, 4	Good
Ehrenreich & Marston, 2019 [57]	Utah Code Sects. 76-7-305	Accessibility, Availability, & Acceptability: Abortion Care	Telemedicine reduced temporal, material, and social barriers to abortion access.	2, 3, 5	Fair
Fuentes et al., 2016 [43]	Texas House Bill 2	Accessibility & Availability: Abortion Care	The closure of clinics in Texas due to restrictive laws resulted in barriers to abortion care, leading to delays and preventing some women from obtaining desired abortions.	1, 3, 5	Fair
Fuerst et al., 2024 [58]	Dobbs v Jackson Women’s Health Organization	Accessibility: Abortion Care	Following the Dobbs decision, 14.3% of abortions in Oregon were out-of-state residents, compared to 9.6% pre-Dobbs.	2, 3	Poor
Garcia and Crosby, 2020 [49]	Oregon Health Plan Medicaid Policy	Accessibility: Gender-Affirming Care	Adolescents described barriers to care despite living in a supportive state, including insurance denials, long waitlists, travel burdens, and difficulties with providers. Although Oregon’s Medicaid expansion improved access for some participants, 20% still had insurance plans that did not cover hormone therapy, and several participants described having to educate their healthcare providers.	1, 3, 5	Good
Jerman et al., 2017 [46]	State Level Abortion Restrictions	Accessibility & Availability: Abortion Care	Participants described traveling across state lines for abortion care because of state restrictions, clinic closures, and limited appointments. Nineteen participants reported delays that resulted in abortions at later gestations, 17 described negative mental health effects, and six considered or attempted to end their pregnancies on their own using methods such as misoprostol, herbs, or physical trauma.	1, 2, 5	Fair
Johnson et al., 2025 [64]	Dobbs v Jackson Women’s Health Organization	Accessibility & Availability: Abortion Care	Women were motivated to obtain advance provision of medication abortion due to concerns about access to abortion under current and future restrictions, particularly following the Dobbs decision.	4	Poor
Kimport & Weitz, 2025 [61]	Fetal Pain-Based Abortion Ban	Accessibility & Quality: Abortion Care	Participants who obtained abortions after 24 weeks described fetal pain and viability laws as strict gestational limits that prevented in-state abortion access and forced travel for care. They reported that providers often denied care based largely on gestational age, including in pregnancies involving serious fetal diagnoses or risks to the woman’s health.	3, 5	Fair
King et al., 2025 [14]	Dobbs v Jackson Women’s Health Organization	Accessibility: Contraception	Among adolescents and young adults, changes in new contraceptive prescriptions and IUD and implant placement services after Dobbs were not statistically significant for either ages 15–18 or ages 19–26. Service use among ages 19–26 increased by an average of 2,621.9 services per month immediately after Dobbs, a change that was nearly statistically significant (*p* = 0.06).	4	Good
Koenig et al., 2023 [56]	Dobbs v Jackson Women’s Health Organization	Accessibility: Abortion Care	Telehealth was more likely to make timely abortion access possible for patients ages 24 years and younger compared with patients ages 35 years and older (48.3% vs. 35.2%; PR 1.4, 95% CI 1.2–1.6).	2	Poor
Kuroki, 2025 [48]	Affordable Care Act	Accessibility & Availability: Abortion Care	Medicaid expansion was associated with lower abortion rates among young adults, with declines ranging from 1.6 to 2.6 abortions per 1,000 women among those ages 18–19 and from 1.1 to 2.3 per 1,000 women among those ages 20–24.	1	Good
Ralph et al., 2024 [53]	Dobbs v Jackson Women’s Health Organization	Accessibility: Abortion Care	Among adolescents who attempted self-managed abortion (SMA), the proportion of adolescents younger than 17 years decreased from 28.6% before to 22.9% after Dobbs. The proportion among those aged 20–24 increased from 22.0% to 24.6 but rates among those ages 18–19 remained relatively stable. LGBTQ+ identity was associated with an increase in SMA attempts following Dobbs compared with before the decision (*p* = 0.03), whereas no significant differences were observed across racial and ethnic groups (*p* > 0.05).	1	Fair
Ramesh et al., 2016 [59]	Illinois Parental Notification Act	Accessibility: Abortion Care	The percentage of youth obtaining first-trimester abortions declined from 5.8% before the law to 4.9% afterward, though not statistically significant, whereas abortion procedures among young adults ages 18–21 increased by 8.8%. For some youth, gestational age at the time of care increased. Most youth met the notification requirement by phone (80.1%), notifying their mother (86.7%).	3	Good
Roberts et al., 2016 [45]	Utah Code Sects. 76-7-305	Accessibility: Abortion Care	Adolescents seeking abortion care reported substantial financial and logistical burdens, which significantly delayed engagement in abortion care. These delays contributed to emotional distress, including feelings of anxiety, uncertainty, stress, and frustration.	1, 3	Good
Strasser et al., 2025 [55]	Dobbs v Jackson Women’s Health Organization	Accessibility: Contraception	Adolescent visits for tubal sterilization and vasectomy increased in states likely to ban abortion, with difference-in-differences analyses showing 6.88 more tubal sterilizations and 3.39 more vasectomies per state per month between May and December 2022 compared with states unlikely to ban abortion. Qualitative findings showed growing considerations for permanent contraception and fear and worries about the safety surrounding pregnancy and reproductive autonomy.	1, 4, 5	Good
Upadhyay et al., 2014 [52]	State Level Gestational Limits	Accessibility: Abortion Care	Gestational limits created substantial barriers and the delay of abortion care, with women reporting issues with travel and procedure costs, delayed pregnancy recognition, insurance problems, not knowing where to obtain care, and not knowing how to reach a provider. Those seeking later abortions were also more likely to travel long distances for care, with some traveling more than 100 miles. Those turned away from care had difficulties receiving care elsewhere.	1, 3	Good
Vear et al., 2023 [47]	Dobbs v Jackson Women’s Health Organization	Accessibility: Abortion Care	Most adolescents reported needing financial support (62%), transportation (42%), time away from work or school (19%), and lodging (13%) as major barriers to access abortion care. Participants also described needing clinical support, including access to providers, abortion information, and insurance, and only 4% reported no barriers to obtaining abortion care.	1, 5	Poor
Washburn et al., 2024 [50]	State Level LGBTQ+ Legislation	Accessibility: Physical & Mental Health Care	Overall, 30.8% of youth did not receive needed physical health care, and 32.6% did not receive needed mental health care, with barriers including lack of knowledge, fear, transportation difficulties, and inability to pay. LGBTQ+ youth experienced greater barriers to care due to policy in comparison to non-LGBTQ+ youth, having more than twice the odds of not receiving needed mental health services (aOR = 2.27), while LGBTQ+ youth assigned female at birth (versus male at birth) had significantly higher odds of not obtaining needed physical health care (aOR = 2.55).	1, 5	Fair
White et al., 2016 [44]	Alabama Administrative Code Rule 420-5-1-0.03	Accessibility & Availability: Abortion Care	Following policy implementation, many adolescents described significant barriers to obtaining abortion care, including traveling long distances, difficulty locating accurate clinic information, clinic scarcity, multiple required visits, lost wages, and arranging transportation or childcare.	1, 3, 5	Fair
White et al., 2026 [60]	Texas Senate Bill 8	Accessibility: Abortion Care	The number of adolescents receiving in-state facility-based abortions decreased from 954 before SB8 to 385 after SB8. Out-of-state abortions among adolescents increased from 62 to 366 during the same period. Total abortions among minors still declined overall, and 64% of adolescents traveling for care went to states with parental notification requirements.	3	Fair

Note. 3AQ Outcome represents Accessibility, Availability, Acceptability, and Quality of health care

*Themes identified: (1) Financial barriers and facilitators to care; (2) Changes in geographic access; (3) Delays in care; (4) Fear, anxiety, and loss of bodily autonomy; (5) Impacts on physical and mental health

### Synthesis

To synthesize the data, we conducted a thematic analysis [41]. The thematic analysis was used to identify patterns across the 25 studies on how the politicized policy, rule, or regulation shaped the right to health (accessibility, availability, acceptability and quality of care) among adolescent patients/clients in the results. As part of the thematic analysis, we categorized the statistical and qualitative results between the policy and right to health into positive, negative, or no associations. We then extended the analysis to define how the policy, rule or regulation changed the right to health and other health outcomes that inductively arose from the studies among the adolescent sample.

## Results

Across the 25 studies, we identified five main themes that emerged examining the relationship of the policy and the right to health: (1) financial barriers and facilitators to care, (2) changes in geographic access, (3) delays in care, (4) fear, anxiety, and loss of bodily autonomy, and (5) impacts on physical and mental health.

1. Financial Barriers and Facilitators to Care

Financial burden emerged as a central mechanism shaping whether, when, and how adolescents and young adults accessed care. Costs extended well beyond the procedure itself, accumulating through travel, lodging, childcare, missed work, and repeat visits that are often mandated by policy [42–45]. As one study noted, “multiple barriers appeared to have a compounding effect,” producing delays and distress that reshape care trajectories [46]. This accumulation was reflected in how patients narrated their experience. Some described these costs extending beyond “not only money at the clinic,” but also viewed additional visits as a “waste of gas money, waste of time away from work” [42, 44]. For adolescents and young adults seeking abortion care, financial need was inseparable from dependence and structural precarity: “I would need money and paid leave to travel out of state” [47]. Even relatively modest expenses could become decisive barriers when layered onto unstable income and limited autonomy.

Insurance coverage can mitigate financial barriers but does not eliminate them [48]. Across abortion care studies, patients without coverage often delayed care while assembling funds for both the procedure and surrounding cost of access [42, 46]. Even when some financial support was available, indirect costs—including transportation, childcare, and missed work—continued to shape access to care [44, 45]. Similar patterns appeared in gender-affirming care, where inclusive insurance facilitates access, but coverage gaps and geographic concentration of providers still required patients to navigate substantial financial and logistical strain [49–51]. Delays in abortion care driven by cost increases gestational age and overall expense, while also pushing some patients towards self-management, continuation of pregnancy, or greater reliance on long-acting or permanent contraception [52–55]. In this way, policy-driven financial burdens operated as barriers that built over time: they accumulate, delay, and ultimately redirect care, with the greatest impact on those with the fewest financial resources [42, 44, 46].

2. Changes in Geographic Access

Legal constraints surrounding abortion and gender-affirming care have precipitated care deserts within regions of the country. In these regions, traveling out of state or implementing novel models of care have been necessary to connect adolescents to gender-affirming and abortion care. Telehealth emerged as a key facilitator of care. One cross-sectional study conducted in California found telehealth to be a reliable service to combat the restrictive legal environment with 43% of patients survey reporting that telehealth made it possible to receive timely abortion care; patients younger than 25 years of age reported telehealth as the most likely facilitator of timely abortion access [56]. Telehealth importantly reduced travel burdens for adolescents and young adults. A participant in one qualitative study described their rationale behind choosing telehealth, stating the following: “I would have to go make a ten-hour drive just to have that little fifteen-minute meeting, in person. And then come back again for the actual procedure. It would have been a big waste, a big, big time-consuming event, if I had to actually go travel to see somebody rather than do [the information visit] over the internet [57].”

Though telehealth improved access to healthcare services, it was not always widely available, creating access barriers related to care. Fuerst et al. (2024) found that the *Dobbs* decision translated to a significant increase in the number of out of state travelers seeking abortion care in Oregon [58]. Furthermore, traveling for services was met with numerous challenges. Another participant in Ehrenreich & Marston’s (2019) qualitative study recounted an unfortunate reality of traveling almost 500 miles for a surgical abortion [57]. This need to travel was especially burdensome given that her pregnancy was the result of sexual assault, emphasizing the psychological impacts of abortion restrictions and the lack of sexual assault exceptions in many states [57]. In the context of gender-affirming care bans, one study examined the drive time to the nearest clinic before and after legislative bans, observing a striking increase in driving time for youth in states that enacted restrictions [51]. For many adolescents traveling to receive care, the logistics of commuting alone presented challenge, such as unsafe weather conditions and lack of access to safe, dependable travel [46].

3. Delays in Care

Across the included studies, policies such as parental notifications, mandatory waiting periods, gestational limits, and restrictions on gender-affirming care consistently led to harmful delays in care. Parental notification policies in Illinois and Texas contributed to a significant decrease in youth younger than 18 receiving abortion care and an increase in gestational age when receiving care [59, 60]. Mandatory waiting periods like Utah’s 72-hour waiting period abortion policy resulted in a median wait time of 8 days and a range of 5 − 17 days from initial visit to abortion appointment [57]. Alabama’s 48-hour waiting period led to only three of 25 participants receiving abortion care within one week of their mandatory initial visit [44]. These “brief waiting periods” were further exacerbated by a lack of policy transparency, introductory visits before scheduling, clinic closures, appointment scarcity, provider biases, travel requirements, and cost [42–45, 57]. Although these laws mandated a wait time of days, the reality was weeks. This delay in care contributed to serious mental distress, and for some participants, the delay meant they reached a clinical threshold in their pregnancy, where the procedures were hazardous, or they were no longer eligible to receive abortion care [44, 45, 57].

Gestational-limit policies serve as hard stops, delaying care or, rather, eliminating access to care entirely. Upadhyay et al. (2014) estimated that these policies, compounded with the lack of healthcare infrastructure contributed to over 4,000 women in the United States carrying unwanted pregnancies to term in 2008 alone [52]. In another study, delay and elimination of care were observed even when participants initiated the proper processes before the gestational limits outlined in Texas’ House Bill 2 policy [43]. Additionally, the overturning of *Dobbs v. Jackson Women’s Health Organization* has contributed to delays in abortion care, as individuals often must travel to other states, increasing wait times and gestational age at the time care is received [58]. Like the waiting-period laws, gestational limits created immense travel and financial burdens and deteriorated the mental health of birthing persons seeking care [43, 52, 58, 61].

Furthermore, policies restricting gender-affirming medical care severely delayed treatment for many transgender and gender-diverse youth and young adults. Carter et al. (2026) found that nearly half of their participants experienced gaps in hormone access and delays in care after Senate Bill 254 passed in Florida prohibiting gender-affirming care for those under 18 [62]. Similarly, the Indiana Senate Enrolled Act 480 forced transgender and gender-diverse youth and their caregivers to decide between an abrupt withdrawal from care, rationing care, accessing care across state lines, or delaying care until youth become of “legal” age [63]. Even in states with broad gender-affirming access, such as Oregon, policies regarding identification and prior authorizations for insurance delayed care [49]. Despite variations in policy focus and state legislative contexts, delays in care remained prevalent across studies examining adolescents right to abortion and gender-affirming care.

4. Fear, Anxiety, and Loss of Bodily Autonomy

Restrictive policies on gender-affirming and abortion care have created great fear and anxiety for adolescents seeking evidence-based care for their mental and physical health. The limitations placed on their ability to make decisions about their bodies have led many to seek advance provision of medications, permanent contraceptive methods, and/or delaying desired care [14, 54, 55, 62–65]. Adolescent perspectives shared pervasive feelings of constraint and loss of bodily autonomy [14, 54, 55, 62–65].

In response to post-*Dobbs* bans and restrictions on abortion care, young adults looked to various methods of contraception to assert control over their reproductive futures [14, 54, 55, 65]. One cross-sectional study found a significant increase in demand for intrauterine devices in counties with longer travel distances to abortion providers [54]. Similarly, two studies found increased support for and motivation to seek advance provision of abortion medication as a backup plan due to abortion restrictions [64, 65]. Some adolescents sought more permanent measures. Visits for tubal sterilization and vasectomy increased significantly for young adults after May 2022, especially in states that were likely to enact bans on abortion [55]. However, the findings of King et al. demonstrated limited changes in adolescent and young adult contraceptive use despite qualitative expressions of fear, loss of agency, and concerns over access, possibly reflecting barriers to access [14].

As a result of restrictive policies on gender-affirming care, transgender and gender-diverse youth have expressed fear and anxiety in respect to governmental control over their healthcare decisions. Participants exhibited anxiety over navigating complex health systems, worry over losing access to gender-affirming hormone therapy, and fear over physical harm due to a hostile social climate [62, 63]. One young transgender woman stated, “…It was a little complicated for me to accept the fact that even though I had just begun with my journey, it may have come to a short end just because of the fact that somebody else is trying to control what I do with my body [62].”

5. Impacts on Physical and Mental Health

Rapidly shifting policy regarding reproductive and transgender healthcare has destabilized essential adolescent care and affected daily health decisions by introducing uncertainty, fear, and risk into already stigmatized care. Individuals seeking abortion care, particularly in states with severe restrictions, reported immediate delays in care leading to negative health outcomes for adolescents seeking abortion care [42, 44, 46, 47]. In these moments of desperation, some contemplated unsafe self-managed abortion, such as use of herbs, use of foreign objects, or self-injury [43, 46, 57, 61]. Others expressed thoughts of suicide, underscoring the psychological toll of these restrictions [43, 57, 61]. Others with high-risk pregnancies found themselves with providers unable or unwilling to offer alternative treatment options, leaving them to continue pregnancies that resulted in complex births with uncertain neonatal outcomes—affecting the health of both the pregnant person and their neonate [61]. Seeing these negative health outcomes, an increasing number of adolescents cite such scenarios as their reason for seeking permanent contraception (i.e. tubal ligation, vasectomy, hysterectomy, etc.), despite knowledge of the impact sterilization can have on long-term health outcomes [55].

LGBTQ+ individuals also found themselves navigating increasingly hostile and confusing healthcare with difficulty finding affirming providers and widespread confusion about what services were still legally available [51, 62, 63]. Transgender and gender-diverse youth were especially affected. Some turned to informal networks, relying on friends or online communities to obtain hormones when clinical access became uncertain or restricted [62, 63]. Others began rationing their medications or altering dosages without medical supervision, increasing the risk of adverse health effects [62, 63]. LGBTQ+ individuals also reported increased anxiety, depression, and fear since policies restricting gender-affirming care went into place [49, 50, 62, 63]. Despite these psychological impacts, many LGBTQ+ adolescents avoided seeking mental health services out of fear of discrimination [50, 63]. Their avoidance is particularly troubling given the increased risk of self-harm and suicide within this population.

## Implications for Care

The findings of this rapid review have important implications for adolescent medicine clinical practices. Providers require institutional support and resources to address increased patient psychosocial concerns and potentially higher patient volume as a result of restrictive legislation [66, 67]. Interdisciplinary care teams with mental health providers and patient navigators can help support providers in addressing these concerns. For those in protected states, they are likely to experience an influx of out-of-state adolescent patients seeking abortion and/or gender-affirming care [47]. Increased administrative support can help support this increased workload. In all settings, medical-legal partnerships that can connect patients and providers with legal expertise and resources have proven to be an important resource [49, 68, 69].

Surprisingly, our review of evidence did not return any studies on the impact of policy on eating disorder care for adolescents given findings in established literature [70–76]. Transgender and gender-diverse adolescents already experience disproportionately high rates of eating disorders, as disordered eating behaviors are often reported coping mechanisms for gender dysphoria [70, 71]. Gender-affirming hormone therapy and surgeries, however, have been shown to improve mental health outcomes and decrease disordered eating behaviors [72]. It appears very likely then that legislation restricting gender-affirming care access could lead transgender and gender-diverse adolescents to increasingly rely on disordered eating behaviors to cope with gender dysphoria. Similarly, legislation restricting abortion care likely exacerbates eating disorders and negative health outcomes for young adults with disordered eating behaviors. Studies have shown that women with anorexia nervosa are more likely to seek abortion care and have significantly higher rates of unplanned pregnancies [73, 74]. At the same time, post-*Dobbs* legislation has been associated with increased rates of anxiety and depression for reproductive-aged women in states with care restrictions [75, 76]. The intersecting nature of reproductive health, eating disorders, and mental health make young women an especially vulnerable population in the post-*Dobbs* era. Providers should be aware of these potential risks for increased disordered eating behaviors in adolescents and recognize the need for future research in this area.

Ultimately, policy advocacy is necessary to change legislation that harms adolescents. This review demonstrates that legislation restricting abortion and gender-affirming care leads to harmful care outcomes for adolescents, including delays in preventative care, limitations on health decision-making, and worsened mental health outcomes. As healthcare providers continue to advocate for adolescents’ right to health, they should look to the connections between reproductive justice and transgender rights; both ultimately assert the right to bodily autonomy [77, 78].

## Future Research Directions

The politicization of abortion and gender-affirming care disproportionately impacts those from underserved communities [79–81]. Many of these communities face barriers to accessing abortion and gender-affirming care due to structural and interpersonal stigma, lack of telehealth services, lack of appointment availability, lack of geographic access, lack of insurance and other financial costs [81–84]. However, the impact of politization on healthcare access for those with multiple marginalized identities was not explored in any of the review’s extracted studies with adolescents and young adults. Future research should focus on exploring these intersectional identities to examine how politization impacts healthcare access related to age, race, ability, income, gender identity, sexual orientation, rural/urban living, and other underserved identities.

## Limitations

This rapid review is not without its limitations. We focused on studies conducted in the United States, which excluded the ways that politicization of health may affect adolescents’ right to health in other parts of the world. While we would normally exclude studies assessed as “poor” during the quality assessment, we decided to include them in this rapid review because this is an emerging and rapidly changing area of study, often including difficult-to-reach populations. We felt it important to include all studies that passed full text review. Lastly, many of these studies do not disaggregate adolescent populations by race, ethnicity, or gender identity not allowing for understanding about sub populations who have scientifically demonstrated inequities.

## Conclusions

Politicized legislation limiting access to reproductive and gender-affirming care has significantly impacted the availability, accessibility, acceptability, and quality of care for adolescents. Restrictive policies created structural barriers, such financial burden and changes in geographic access, that led to negative health outcomes, including delays in care, worsened mental health, and loss of bodily autonomy.

## Supplementary Information

Below is the link to the electronic supplementary material.

Supplementary Material 1 (XLSX 26.5 KB)

## Data Availability

All data supporting the findings of this study are available within the paper and its Supplementary Information. The extracted data on study characteristics for the 25 articles are found in Table 1, and the extracted results and quality ratings are in Table 2.
